# Evidence for Soft Selective Sweeps in the Evolution of Pneumococcal Multidrug Resistance and Vaccine Escape

**DOI:** 10.1093/gbe/evu120

**Published:** 2014-06-10

**Authors:** Nicholas J. Croucher, Claire Chewapreecha, William P. Hanage, Simon R. Harris, Lesley McGee, Mark van der Linden, Jae-Hoon Song, Kwan Soo Ko, Herminia de Lencastre, Claudia Turner, Fan Yang, Raquel Sá-Leão, Bernard Beall, Keith P. Klugman, Julian Parkhill, Paul Turner, Stephen D. Bentley

**Affiliations:** ^1^Center for Communicable Disease Dynamics, Department of Epidemiology, Harvard School of Public Health, Boston, Massachusetts; ^2^Pathogen Genomics, The Wellcome Trust Sanger Institute, Wellcome Trust Genome Campus, Hinxton, Cambridge, United Kingdom; ^3^Respiratory Diseases Branch, Centers for Disease Control and Prevention, Atlanta, Georgia; ^4^Institute for Medical Microbiology, National Reference Center for Streptococci, University Hospital, RWTH Aachen, Aachen, Germany; ^5^Samsung Medical Centre, Sungkyunkwan University School of Medicine and Asia Pacific Foundation for Infectious Disease, Seoul, South Korea; ^6^Department of Molecular Cell Biology, Sungkyunkwan University School of Medicine, Suwon, South Korea; ^7^Laboratory of Molecular Genetics, Instituto de Tecnologia Química e Biológica, Universidade Nova de Lisboa, Oeiras, Portugal; ^8^Laboratory of Microbiology, The Rockefeller University, New York, New York; ^9^Shoklo Malaria Research Unit, Mahidol Oxford Tropical Medicine Research Unit, Faculty of Tropical Medicine, Mahidol University, Mae Sot, Thailand; ^10^Mahidol Oxford Tropical Medicine Research Unit, Faculty of Tropical Medicine, Mahidol University, Bangkok, Thailand; ^11^Centre for Tropical Medicine, Nuffield Department of Medicine, University of Oxford, United Kingdom; ^12^Institute of Antibiotics, Huashan Hospital, Fudan University, Shanghai, China; ^13^Hubert Department of Global Health, Rollins School of Public Health and Division of Infectious Diseases, School of Medicine, Emory University; ^14^Centre for Respiratory Diseases and Meningitis, National Institute for Communicable Diseases, Gauteng, South Africa; ^15^Department of Medicine, University of Cambridge, Addenbrooke’s Hospital, United Kingdom

**Keywords:** bacterial evolution, recombination, vaccine escape, antibiotic resistance, selective sweeps, phylogenomics

## Abstract

The multidrug-resistant *Streptococcus pneumoniae* Taiwan^19F^-14, or PMEN14, clone was first observed with a 19F serotype, which is targeted by the heptavalent polysaccharide conjugate vaccine (PCV7). However, “vaccine escape” PMEN14 isolates with a 19A serotype became an increasingly important cause of disease post-PCV7. Whole genome sequencing was used to characterize the recent evolution of 173 pneumococci of, or related to, PMEN14. This suggested that PMEN14 is a single lineage that originated in the late 1980s in parallel with the acquisition of multiple resistances by close relatives. One of the four detected serotype switches to 19A generated representatives of the sequence type (ST) 320 isolates that have been highly successful post-PCV7. A second produced an ST236 19A genotype with reduced resistance to β-lactams owing to alteration of *pbp1a* and *pbp2x* sequences through the same recombination that caused the change in serotype. A third, which generated a mosaic capsule biosynthesis locus, resulted in serotype 19A ST271 isolates. The rapid diversification through homologous recombination seen in the global collection was similarly observed in the absence of vaccination in a set of isolates from the Maela refugee camp in Thailand, a collection that also allowed variation to be observed within carriage through longitudinal sampling. This suggests that some pneumococcal genotypes generate a pool of standing variation that is sufficiently extensive to result in “soft” selective sweeps: The emergence of multiple mutants in parallel upon a change in selection pressure, such as vaccine introduction. The subsequent competition between these mutants makes this phenomenon difficult to detect without deep sampling of individual lineages.

## Introduction

*Streptococcus pneumoniae* (the “pneumococcus”) is an oronasopharyngeal commensal bacterium and respiratory pathogen representing a common cause of pneumonia, bacteremia, and meningitis. Global estimates suggest that the pneumococcus was responsible for 826,000 deaths in children under 5 years in 2000 (O'Brien et al. 2009). A major clinical concern over recent decades has been pneumococcal multidrug resistance (MDR), defined as resistance to β-lactams and at least two other classes of antibiotic, the first example of which was detected in 1977 (Jacobs et al. 1978). Genotyping of MDR pneumococci has suggested that many such isolates belong to a small number of internationally disseminated “clones” of closely related bacteria (Klugman 2002). It was hoped that their success would be reversed by the heptavalent antipneumococcal polysaccharide conjugate vaccine (PCV7), which targeted the seven “vaccine-type” pneumococcal capsule types (directly corresponding to serotypes) accounting for the majority of pre-PCV7 β–lactam-resistant isolates from invasive pneumococcal disease in the United States (Whitney et al. 2000).

Although the incidence of antibiotic-resistant disease has typically decreased following PCV7’s introduction, owing to the elimination of vaccine serotypes, the relative prevalence of antibiotic resistance in the pneumococcal population has generally not fallen dramatically (Kyaw et al. 2006; Huang et al. 2009). This is partly a consequence of particular MDR clones being associated with multiple capsule types likely as a consequence of “serotype switching”: The acquisition of a novel capsule type through exchange of sequence at the capsule polysaccharide synthesis (*cps*) locus, which determines the serotype. Such a process allows clones generally associated with vaccine serotypes to persist in the post-PCV7 environment in the form of mutants expressing nonvaccine type capsules (Beall et al. 2011; Hanage, Bishop, Huang, et al. 2011; Simões et al. 2011).

In the years following PCV7’s introduction in the United States, the clone in which serotype switching had the biggest impact was Taiwan^19F^-14, also known as PMEN14. This clone was originally detected as having the vaccine-type 19F capsule when first characterized by applying multilocus sequence typing (MLST) (Aanensen and Spratt 2005) to isolates from a Taiwanese hospital in 1997 (Shi et al. 1998). These multilocus sequence type (ST) 236 isolates were found to be resistant to β-lactams, tetracyclines, and macrolides. Epidemiological surveillance subsequently identified closely related serotype 19F isolates in other parts of Southeast Asia (Bogaert et al. 2002; Ip et al. 2002), South Africa (McGee et al. 2001), and the United States (Corso et al. 1998). Isolates that appear to be members of this clone, based on genotyping information, but expressing the nonvaccine serotype 19A have become similarly widespread. In Southeast Asia, in the late 1990s, 19A isolates were detected of ST320; these represented a double locus variant (DLV) of ST236, as the two STs shared identical alleles at five of the seven MLST loci (Farrell et al. 2004; Ko and Song 2004; Choi et al. 2008). Subsequently, ST320 has become a highly prevalent multidrug-resistant genotype post-PCV7 in surveys of carriage (Hanage, Bishop, Lee, et al. 2011) and in cases of invasive pneumococcal disease (Moore et al. 2008; Beall et al. 2011) in the United States. The 19A capsule type was also found in isolates with STs 271 (a single locus variant, or SLV, of ST236; these STs are identical at six of the seven MLST loci) and 236 itself. In this latter case, isolates were found to be less resistant to β-lactams than most PMEN14 isolates. Along with the observation of sequence similarity to a putative donor, this led to the hypothesis that a capsule-switching recombination had also altered the linked penicillin-binding protein (PBP) genes that determine susceptibility to such antibiotics (Moore et al. 2008).

STs 236, 271, and 320 are all grouped within clonal complex (CC) 320, which also includes non-MDR pneumococci. However, this genotyping information is not sufficient to precisely reconstruct the pattern by which the MDR phenotype, and vaccine escape 19A isolates, emerged. MDR may have emerged once, in which case PMEN14 would represent a single lineage within which subsequent diversification and intermittant reversion to antibiotic susceptibility may have contributed to the observed diversity. Alternatively, resistance may have been acquired on multiple occassions by closely related bacteria, in which case the diversity would represent the convergent evolution of susceptible progenitors. Such an observation would suggest that some aspect of the genotype may predispose it toward becoming a successful MDR pneumococcus.

Phylogenomic analysis can provide the necessary resolution to distinguish these alternative hypotheses when isolates of different phenotypes are placed in the context of a broader sample. Alongside the isolates representing the variation in antibiotic resistance and serotype, primarily recovered from cases of disease, the collection assembled for this study includes CC320 isolates from a survey of carriage in the Maela refugee camp in Northwestern Thailand (Turner et al. 2012). CC320 isolates were found to be the most common genotype in Maela by an independent population-wide survey (Chewapreecha et al. 2014); hence, this location is able to provide a “snapshot” of the clone’s overall diversity. These isolates also provide a useful comparison as a bacterial population not directly subject to selection by vaccine-induced immunity. This overall collection of bacteria should therefore be able to distinguish between alternative explanations as to the emergence of MDR and subsequent instances of vaccine escape in this set of a clinically important pneumococci.

## Materials and Methods

### Phylogenomic Analysis

DNA samples were collected for all isolates of CC320 available from the sources listed in supplementary table S1, Supplementary Material online. Samples were sequenced on the Illumina GAII and HiSeq platforms (supplementary table S1, Supplementary Material online) as multiplexed libraries. Short read data were then aligned against the *S. pneumoniae* Taiwan^19F^-14 genome (Donati et al. 2010) (EMBL accession code: CP000921) using SMALT v0.6.4 to generate a multiple genome alignment, from which polymorphic sites were identified, as described previously (Croucher et al. 2012). Only samples with a mean coverage above 25-fold and unambiguously calling bases at >90% of the positions in the reference sequence were used in the analysis. Samples were also excluded if they showed signs of contamination, based on frequency of sites with evidence of multiple alleles, or if their serotype and ST (determined as described previously; Croucher et al. 2011) both significantly deviated from previously determined epidemiological information (i.e., if isolates were of a different serogroup and their MLST profile differed at two loci or more).

Isolate 41_PMEN14 represents an independent culture of isolate TW31, from which the reference sequence was generated. Furthermore, sequences 8561-06 and LMG87 were both generated from independent cultures of the same isolate, as were 7848-05 and LMG95. Prediction of recombinant sequence and generation of a maximum-likelihood phylogeny were then conducted as described in Croucher et al. (2011). In this analysis, all three pairs of sequences from the same isolate were found to be closely related sister leaf nodes in the phylogeny. The same alignment was also analyzed with BRATNextGen (Marttinen et al. 2012), assuming four clusters, using a learned value of alpha, a window size of 1 kb, and a significance threshold *P* value of 0.05 (as calculated from 100 permutations).

A Bayesian coalescent analysis was performed on a subset of the alignment corresponding to the PMEN14 clade using BEAST (Drummond et al. 2012). Base substitutions predicted to have been introduced through recombination were excluded from the alignment used in this analysis. The topology of the phylogeny was fixed as that of the rooted subtree from the overall analysis, with the years of isolation listed in supplementary table S1, Supplementary Material online, used to establish a molecular clock based on a general time reversible substitution model. A relaxed lognormal clock prior (Drummond et al. 2006) was used for the substitution rate and a skyline plot prior (Drummond et al. 2005) was used for the population demography. All values were estimated with an effective sample size of over 200.

### Accessory Genome Distribution

For the analysis of *cps* loci and integrative and conjugative elements (ICEs), Illumina sequence reads were assembled de novo using Velvet (Zerbino and Birney 2008), with scaffolds generated using SSPACE (Boetzer et al. 2011) and sequence improvement conducted using the PAGIT pipeline (Swain et al. 2012). The serotype 19A *cps* loci displayed in supplementary figure S5, Supplementary Material online, have been submitted to the ENA with acccession codes HG799504 (isolate 7848-05), HG799505 (isolate 8312-05), and HG799488 (isolate SN28652). The ICEs displayed in supplementary figure S6, Supplementary Material online, have been submitted to the ENA with accession codes HG799503 (ICE*Sp*SPN28652), HG799502 (ICE*Sp*PT814), and HG799501 (ICE*Sp*6027). Nucleotide sequence comparisons were performed using BLAT (Kent 2002) with default settings and analyzed using ACT (Carver et al. 2008).

The distribution of antibiotic resistance genes shown in figure 4 reflects the mapping of sequence reads to the displayed reference sequences identifed in the analysis of the PMEN1 lineage (Croucher et al. 2011) using BWA v0.7.3 (Li and Durbin 2009). The coverage plots were then generated using Samtools (Li et al. 2009) and standardized by dividing the coverage at each base by the number of million reads generated in the sequencing of the sample. Biopython (Cock et al. 2009) was then used to display these data as heatmaps.

**Fig. 1.—:**
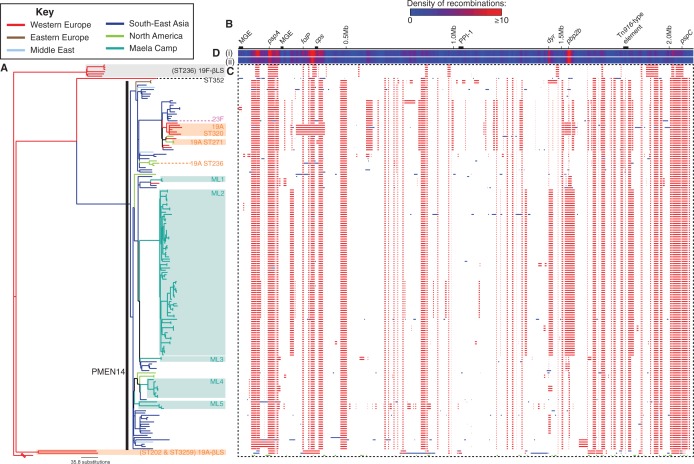
Phylogenomic analysis of CC320. (*A*) Maximum-likelihood phylogeny constructed using the vertically inherited point mutations occurring in the sampled taxa. The tree is colored according to the location in which the isolate was collected, reconstructed through the tree using a maximum parsimony approach. The broken branch is that to the outgroup isolate, SN4691. The majority of the isolates belong to the clade labeled PMEN14, with the other ten isolates split into the labeled “19F-βLS” clade, “19A-βLS” clade, and the “ST352” representative. The blue shaded boxes represent five clades of isolates from the Maela refugee camp (labeled ML1-5). Isolates that have undergone serotype switches with the PMEN14 clade are marked as “23F,” “19A ST271,” “19A ST236,” and “19A ST320.” (*B*) Annotation of the *Streptococcus pneumoniae* Taiwan^19F^-14 reference genome. MGEs and the Pneumococcal Pathogenicity Island-1 genomic island are labeled. Antigen-encoding loci (*pspA*, *pspC*, and the capsule polysaccharide synthesis, or *cps*, locus) are marked, as are genes involved in antibiotic resistance: *pbp2b, dyr*, *folP*, and the Tn*916*-type element (see text). The *pbp2x* and *pbp1a* genes are found shortly upstream and downstream of the *cps* locus, respectively. (*C*) This panel contains a column for each base in the reference sequence, and a row for each taxon in the phylogeny. Colored blocks indicate putative recombination events: These are blue, when the recombinations are reconstructed as having occurred on a terminal branch, and are therefore unique to one isolate, or red, when the event is reconstructed as having occurred on an internal branch, and therefore the recombination is shared by multiple isolates through common descent. (*D*) The density of these independent events is summarized by two heatmaps above the panel, which range from dark blue (where no recombinations occur) to red (indicating sites affected by ten or more recombinations). Heatmap i) reflects the data across the entire collection, whereas ii) summarises only the recombinations occurring within the PMEN14 clade.

### Assessment of Potential Sequence Donors

The regions corresponding to the serotype switching recombinations importing the 19A capsule into SN28652 (ST320) and 8312-05 (ST236) were extracted from de novo assemblies. These sequences were then used to search the de novo assemblies of the serotype 19A ST199 isolates from a study of isolates from Massachusetts (Croucher, Finkelstein, et al. 2013) using Basic Local Alignment Search Tool (BLAST) (Altschul et al. 1990). The best matching isolates were taken as potential sequence donors, and combined with the recipients and all complete publicly available genome sequences (although using only *S. pneumoniae* OXC141 as a representative of the CC180 clade; Croucher, Mitchell, et al. 2013) to represent the species-wide variation in the region flanking the *cps* locus. The regions orthologous to that between SPT_0362 and SPT_0391 (*dexB*) in *S. pneumoniae* Taiwan^19F^-14 (the 31,305 bp 19A Recombinant Region) were extracted from each genome, aligned using MUGSY (Angiuoli and Salzberg 2011) and a phylogeny generated using RAxML (Stamatakis et al. 2005). The same procedure was used to generate the phylogeny for the 31,850 bp control region, defined by the boundaries of coding sequences (CDSs) SPT_0438 and SPT_0473 in *S. pneumoniae* Taiwan^19F^-14.

Antibiotic minimum inhibitory concentrations (MICs) listed in supplementary table S1, Supplementary Material online, were provided by the organizations that originally collected the isolates. For a direct comparison, the penicillin MICs of the isolates listed in table 1 were retested using an *E* test in the same laboratory.

**Table 1:** MICs of Isolates Acquiring 19A Capsules

Strain	Serotype	ST	Penicillin MIC (μg/ml)
7535-06	19F	236	1
8312-05	19A	236	0.25
SN28306	19F	320	4
SN39039	19A	320	4

Note.—These isolates are two pairs, each containing a serotype 19A isolate generated through capsule switching and the serotype 19F isolate most closely related to the ancestral sequence prior to the capsule switching recombination. In the ST236 isolates, the recombination at the capsule locus leads to a fall in penicillin resistance, because the flanking penicillin-binding protein genes are replaced by alleles similar to those in penicillin-susceptible isolates. This is not the case in ST320, where the level of resistance is maintained after the acquisition of the 19A capsule.

### Individual Gene Analyses

Sequences corresponding to the resistance genes *pbp1a*, *pbp2x*, *pbp2b*, *dyr,* and *folP* were extracted from de novo Velvet (Zerbino and Birney 2008) assemblies of the CC320 isolates. The protein sequences were aligned using MUSCLE (Edgar 2004) and then backtranslated to give a codon alignment. This was then analyzed using BAPS (Tang et al. 2009) to provide an estimate of the number of different alleles in the collection, which was then used to inform an analysis of the alignment using BRATNextGen (Marttinen et al. 2012) with alpha fixed at 20 and a window length of 100 bp. Recombinant segments were identified using a threshold *P* value of 0.05, as calculated from 100 permutations.

## Results

### PMEN14 Is a Rapidly Recombining Lineage

The sample collection consisted of 175 sequence data sets from 173 representatives of CC320 isolated between 1997 and 2009, containing examples of serotypes 19F, 19A, and 23F. They originated in 12 countries, the majority coming from Southeast Asia but also including representatives from the Middle East, Europe, and the United States. All isolates were sequenced as multiplexed libraries using the Illumina platform, generating paired-end reads as detailed in supplementary table S1, Supplementary Material online. Read pairs were aligned against the complete reference genome of *S. pneumoniae* Taiwan^19F^-14, representing isolate TW31 from the original identification of the clone in Taiwan in 1997 (Donati et al. 2010), and bases called using previously defined criteria (Harris et al. 2010). Resequencing of TW31 identified ten base substitutions relative to the reference genome. Similarly, for two isolates sequenced in duplicate in this study, one of serotype 19A and the other 19F, the pairs of consensus sequences were only distinguished by two and three polymorphic sites, respectively. The most divergent isolate was found to be SN4691 of ST1584 (a DLV of ST236), which was used to root the phylogeny. Excepting this sequence, 46,377 polymorphic sites were identified. This alignment relative to the reference was analyzed using an iterative algorithm to simultaneously identify recombinant sequence and construct a phylogeny based only on vertically inherited point mutations in the “clonal frame” of the genome, as described previously (Croucher et al. 2011) (fig. 1). BRATNextGen (Marttinen et al. 2012) produced similar results when applied to the same alignment (supplementary fig. S1, Supplementary Material online).

**Fig. 2.—:**
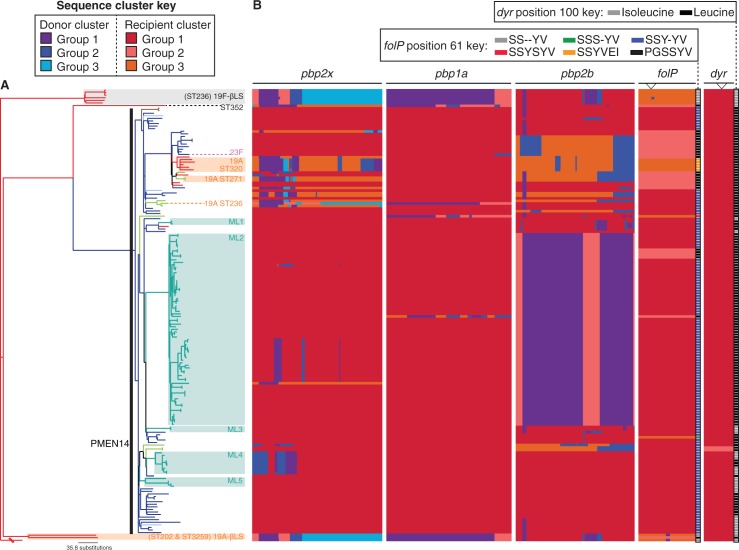
Alterations in sequences of genes associated with resistance. (*A*) The whole genome maximum-likelihood phylogeny is displayed as in figure 1. (*B*) Each column corresponds to a codon alignment of a gene whose sequence influences the antibiotic resistance profile of the host bacterium, with the width of the column representing the length of the alignment. The sequences are arranged according to the class of antibiotics to which they cause resistance: *pbp1a*, *pbp2x*, and *pbp2b* determine resistance to β-lactams, whereas the sequences *dyr* and *folP* determine resistance to trimethoprim and sulphonamides. Each of these alignments were independently analyzed using BRATNextGen, which clusters the sequences into alleles and identifies recombinations. The background color of each row represents the “recipient” cluster to which the overall sequence belongs, with changes in color indicating recombination breakpoints resulting from import of sequence from “donor” clusters. The specific sites of changes within the *folP* and *dyr* genes that are associated with resistance are indicated by the downwards-pointing arrows at the top of the columns. Sulphamethoxazole resistance arises through insertions shortly after S61 in *folP*. The ancestral form of the sequence (S61S–YV) is found in isolates with a grey box to the right of the gene sequence analysis; different colors indicate the distribution of five different alleles encoding insertions in this region of the protein. In the case of *dyr*, trimethoprim resistance is associated with the substitution of isoleucine with leucine at position 100; the amino acid present at this site in each gene is indicated by the black and grey boxes adjacent to the analysis of gene sequences.

The rooted phylogeny suggested that some of the β–lactam-sensitive isolates represented the ancestral phenotype from which the MDR isolates emerged. These susceptible isolates were split into two clades: ST236 isolates of serotype 19F (predicted to be the the ancestral serotype) formed clade “19F-βLS,” and serotype 19A ST202 and ST3559 isolates formed clade “19A-βLS.” The β–lactam-resistant genotypes were monophyletic; however, a single ST352 isolate (an SLV of ST236) was an outlier to the robustly supported PMEN14 clade itself (supplementary fig. S2, Supplementary Material online). Within the PMEN14 clade, 61,096 base substitutions were reconstructed as having occurred across 38,744 polymorphic sites. Of the base substitutions, 58,420 (96%) were imported by 451 recombinations, estimating the per site *r*/*m* statistic (the ratio of base substitutions accumulated by recombination relative to the number of point mutations) as 21.8. This value is far higher than that calculated for PMEN1 using the same approach (7.2), partly due to the larger number of recombinations per point mutation: 0.17 for PMEN14 as compared with 0.10 for PMEN1 (Croucher et al. 2011). Many recombinations were clustered at high densities around the *cps* locus, which included those causing changes of serotype, and the *pspA* gene, which encodes the pneumococcal surface protein A antigen and was affected by 16 putative recombinations. However, the locus encoding the *pspC* antigen, which like *pspA* is observed to frequently undergo recombination in other lineages (Croucher et al. 2011; Croucher, Finkelstein, et al. 2013; Chewapreecha et al. 2014), was only affected by four recombinations. This may be related to the locus being atypical in having two *pspC* paralogues in tandem (Iannelli et al. 2002), an arrangement conserved in all isolates in the collection. Additionally, the loci annotated as mobile genetic elements (MGEs) in the reference sequence contributed little to the apparent diversification. Hence, the per site *r*/*m* statistic only fell to 21.7 when recombinations occurring in such regions were excluded, although this is still lower than the same metric calculated from a smaller independent sample of the same genotype (34.1) (Croucher, Finkelstein, et al. 2013). These remaining sequence exchanges outside of MGEs are likely homologous recombinations, and followed a similar exponential length distribution to that observed in PMEN1 (rate parameter of 1.16 × 10^−^^4^ per bp; 95% confidence interval: 1.07 × 10^−^^4^ to 1.27 × 10^−^^4^ per bp; supplementary fig. S3, Supplementary Material online) (Croucher et al. 2012).

The *r*/*m* ratio also reflected the low number of point mutations (2,676 mutations affecting 2,373 sites) within the PMEN14 clade. A root-to-tip distance plot of genetic divergence over time (*n* = 164, *R*^2 ^= 0.40, *P* value < 2.2 × 10^−^^16^; supplementary fig. S4, Supplementary Material online) provided significant evidence of a molecular clock. A Bayesian coalescent analysis (Drummond et al. 2006) indicated that the lineage arose around 1987 (95% credibility interval: 1981–1991), with a base substitution rate just under four base substitutions per year (1.80 × 10^−^^6^ substitutions/site/year; 95% credibility interval: 1.27 × 10^−^^6^ to 2.20 × 10^−^^5^), only slightly greater than previously calculated values (Croucher et al. 2011; Croucher, Finkelstein, et al. 2013). That the *r*/*m* is high despite this elevated mutation rate emphasizes how quickly PMEN14 acquires material through transformation, resulting in a diverse set of genotypes circulating globally.

### Development of Resistance through Transformation

These extensive levels of homologous recombination led to the import of sequences causing resistance to antibiotics (fig. 2). Resistance to trimethoprim most often arises from I100L substitutions in the Dyr (or FolA) protein (Adrian and Klugman 1997). PMEN14 appears to have originally been trimethoprim sensitive and subsequently imported this resistance mutation on at least ten occasions, each time associated with one of the 15 recombinations that affects the *dyr* gene. The mutation has also been gained through recombination by the 19A-βLS isolate PT814, which has additionally acquired a *folP* allele with a small insertion at the S61 position, a modification associated with sulphamethoxazole resistance (Maskell et al. 1997). The other two acquisitions of sulphamethoxazole-resistant *folP* alleles seen in the collection correspond to the divergence of the ST352 isolate and the emergence of PMEN14. The continuing diversification of FolP within PMEN14 is clearly supported by the diversity of resistance-associated insertions in the protein, resulting in *folP* (like *dyr*) being a “hotspot” of recombination (fig. 1).

**Fig. 3.—:**
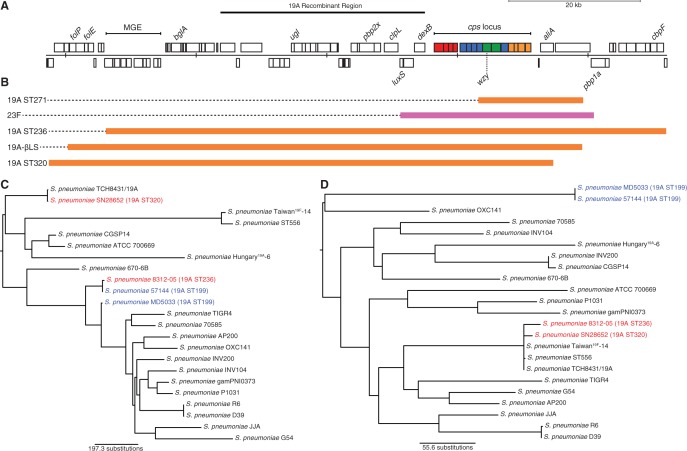
Recombinations causing capsule switching. (*A*) Annotation of the *cps* locus of *S. pneumoniae* Taiwan^19F^-14. Putative CDSs on the forward and reverse strand are annotated above and below the central bar, respectively. The penicillin-binding protein genes, as well as *dexB* and *aliA* that flank the capsule biosynthesis genes, are marked. Within the *cps* locus, regulatory genes are colored red, genes for the biosynthesis of monomeric units are colored blue, genes involved in the polymerization and export of the capsule polysaccharide are colored green, and genes for the production of rhamnose are colored orange. The extent of the 19A Recombinant Region, used to assess potential donors, is marked. (*B*) Predicted homologous recombinations causing changes in capsule type, displayed relative to the annotation shown in (*A*). Each bar, colored by the serotype of the sequence donor, represents the extent of the putative recombination causing the serotype switches annotated in figure 1. The recombination that resulted in the acquisition of the type 23F capsule biosynthesis genes spanned the entire *cps* locus but did not affect *pbp2x*, and only overlapped with a short region at the 3′ end of *pbp1a*. The acquisition of the 19A capsule by ST271 only spans part of the *cps* locus, but still causes a change in serotype. The other three events importing the 19A capsule type are much more extensive. The recombinations in ST320 and the outgroup both encompass *pbp2x* but leave *pbp1a* intact, while the event generating the ST236 19A isolate replaces both *pbp2x* and *pbp1a*. (*C*) Evaluating potential donor sequences. Maximum-likelihood phylogenies for individual sequence segments were generated using an alignment of all publically available complete pneumococcal genomes, along with sequences extracted from the de novo assemblies of ST320 19A isolate SN28652 and ST236 19A isolate 8312-05 (both red) and potential ST199 19A sequence donors (blue). This tree shows the relationships between isolates in the 19A Recombinant Region, predicted to have been imported along with the *cps* locus in ST320 and ST236. This shows that the ST236 19A isolate is very close to the putative donor in this phylogeny, indicating such an exchange may have occurred. However, this is not the case for the ST320 isolate. (*D*) This represents the equivalent phylogeny for the “control region,” immediately downstream of the serotype switching recombinations on the opposite side of the *cps* locus. In this phylogeny, all of the PMEN14 sequences are monophyletic.

Resistance to β-lactams in clinical pneumococcal isolates results from modification of PBPs through incorporation of heterospecific sequence in the *pbp1a*, *pbp2x*, and *pbp2b* genes (Dowson et al. 1993; Sibold et al. 1994). The evolutionary reconstruction (fig. 1) and analysis of gene sequences (fig. 2) suggested that penicillin resistance was independently acquired by PMEN14 and ST352. Since the last common ancestor of both these genotypes, ST352 appears to have accumulated 31 recombinations encompassing 218 kb, including events affecting *pbp1a*, *pbp2b*, and *pbp2x* (but not *murM* or *murN*). Similarly, on the other branch leading from the common ancestor of all the MDR isolates to the last common ancestor of the PMEN14 clade, 35 recombinations affecting 279 kb were predicted to have occurred. The *murMN* genes were once more unaffected, whereas the *pbp1a*, *pbp2x*, and *pbp2b* genes were each again altered through the import of sequence and subsequently continued to diversify throughout the PMEN14 clade.

**Fig. 4.—:**
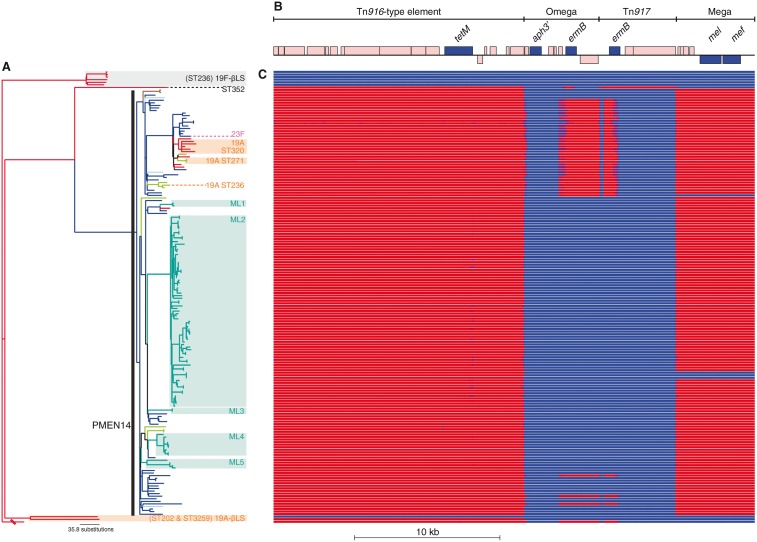
Acquisition and persistence of elements carrying antibiotic resistance genes. (*A*) The whole genome phylogeny as displayed in fig. 1. (*B*) The annotation of the concatenated reference sequences of Tn*916*, carrying the *tetM* tetracycline resistance gene, and the Omega, Tn*917*, and mega macrolide resistance cassettes. CDSs associated with the mobility and replication of the conjugative element are colored pink, with the position above or below the central line representing whether they are found on the forward or reverse strand of the sequence. Genes conferring resistance to antibiotics are colored blue and labeled. (*C*) Sequence read mapping heatmap. This panel contains one row for each isolate, which ranges from blue, where there is no mapping of sequence reads, to red, where there is high mapping of sequence reads. This shows that although Tn*916* is conserved among all PMEN14 representatives (while being absent in the majority of the outgroup), the complement of macrolide resistance cassettes is variable within the clade.

### Multiple Instances of Vaccine Escape

Changes in *pbp2x* and *pbp1a* are often associated with changes in serotype (fig. 2) as they flank the *cps* locus. Five serotype switches were identified, one of which involved the acquisition of the type 23F capsule, another antigen included in the PCV7 vaccine. This recombination extended over 29.6 kb of the reference sequence and almost entirely preserved the sequences of *pbp1a* and *pbp2x* (fig. 3). The other four switches resulted in the acquistion of the 19A serotype, not targeted by the PCV7 vaccine. One of these, a 78.8-kb long exchange that replaces *pbp2x* as well as an extensive upstream tract, caused the serotype switch characteristic of clade 19A-βLS. However, only five amino acid substitutions were introduced into the 750 aa Pbp2X protein, none of which altered the β-lactam sensitivity of the isolates.

Within the PMEN14 clade, the shortest capsule switching recombination (16.1 kb relative to the reference sequence) imported the 19A capsule into ST271. Although this import failed to span the entire *cps* locus, it did affect the *wzy* polymerase gene, thought to be the crucial determinant distinguishing the 19F and 19A serotypes (Mavroidi et al. 2007). The resulting mosaic *cps* locus within this post-PCV7 vaccine-escape lineage, represented by a pair of sequences corresponding to an isolate from Minnesota in 2005, therefore appeared to be a mosaic of the ancestral sequences characteristic of serotype 19F in the 5′ region with an imported *wzy* gene characteristic of serotype 19A (supplementary fig. S5, Supplementary Material online). A separate, but nearby, recombination occurring on the same branch of the phylogeny changed the *pbp2x* sequence without affecting β-lactam resistance. The *pbp2x* gene was also affected directly by the other two, longer, recombinations that imported the 19A capsule type into PMEN14. One, associated with the emergence of the highly resistant ST320 clade, replaced one resistant *pbp2x* allele with another. However, the recombination that occurred in the ST236 19A isolate *S. pneumoniae* 8312-05 was the longest at 85.8 kb relative to the reference and replaced both *pbp2x* and *pbp1a* with alleles similar to those of the sensitive outgroup isolates (fig. 2). In concordance with previous observations (Moore et al. 2008), testing revealed that *S. pneumoniae* 8312-05 had increased susceptibility to β-lactams (table 1).

The ST199 lineage has been suggested to be the source of the 19A *cps* locus in both ST236 (Moore et al. 2008) and ST320 (Pillai et al. 2009). To test these hypotheses, a phylogeny was generated for the 19A Recombinant Region, representing an approximately 40-kb stretch of the genome immediately upstream of the *cps* locus that is predicted to have been imported along with the *cps* locus in both the ST320 and ST236 switches (fig. 3). In the case of ST236, this was found to be very similar to a potential ST199 donor (Croucher, Finkelstein, et al. 2013), although such a match could not be found for the ST320 isolate. An equivalently long stretch of sequence immediately downstream of the *cps* locus, unaffected by either serotype switching recombination, was used as a control. In this locus, both 19A isolates were most similar to the reference genomes of the PMEN14 genotype, as expected if they were unaffected by recent horizontal import of sequence. These data are consistent with the hypothesis that the ST236 switch involved a donor of ST199.

### Import of Resistance Cassettes

Heterogeneity in antibiotic resistance profiles was also generated by the movement of MGEs. Tetracycline and macrolide resistance genes were acquired on both branches on which β-lactam resistance emerged (fig. 4). In both cases, this was the result of the acquisition of a Tn*916*-type ICE, carrying a *tetM* tetracycline resistance gene, into which a macrolide resistance cassette had inserted. In PMEN14, the ICE was inserted shortly downstream of the *rpoBC* operon and carried a mega element that encoded a *mef*/*mel* macrolide efflux pump (Del Grosso et al. 2006). In ST352, the Tn*916*-type element was inserted downstream of *lytA*, which encodes the principal pneumococcal autolysin, and carried a Tn*917* macrolide resistance cassette (Shaw and Clewell 1985). This suggests that ST352 and PMEN14 independently acquired these related MGEs. A further acquisition of a Tn*916*-type ICE was observed in the 19A-βLS isolate *S. pneumoniae* PT814, where the transposon was carried within a larger Tn*5252*-type ICE (Ayoubi et al 1991) inserted downstream of the *zmpA* gene (supplementary fig. S6, Supplementary Material online).

The presence of mega within PMEN14 since its inception contrasts with PMEN1 (originating around 1970) (Croucher et al. 2011), likely reflecting macrolide resistance becoming common in pneumococci after the emergence of PMEN1 but before the emergence of PMEN14 (Appelbaum 1992; Baquero et al. 2002). Nevertheless, PMEN14 appears to have also acquired the *ermB* macrolide resistance gene within the Omega cassette (Croucher et al. 2011) on four occasions, resulting in a “Tn*2010*” structure (Del Grosso et al. 2007). One of these instances has persisted through the clade in which the three serotype switches to 19A occurred within PMEN14 in this collection. As well as these instances of resistance emerging, the *mel*/*mef* pump appears to have been deleted twice. One of these deletions was represented by an isolate carrying an *ermB* resistance gene, whereas in the other the loss led to a clade of three isolates becoming sensitive to macrolides (supplementary table S1, Supplementary Material online). These isolates were from the Maela refugee camp (Chewapreecha et al. 2014) and formed part of a large clade of ST4414 isolates (labeled ML2 in fig. 1) that appeared to represent the dissemination of a single clone within the camp.

### Diversity in a Single Unvaccinated Community

The ML2 clade of 80 isolates was calculated as having an *r*/*m* of 12.8, demonstrating that PMEN14’s high level of sequence import is also observed among cocirculating isolates in the absence of vaccination. Furthermore, the overall population of bacteria from Maela was polyphyletic with respect to the rest of the collection (five clades labeled ML1-5 in fig. 1), indicating that the clone has entered the camp at least five times. Based on this minimum, and the dates of isolation, each of the five clades seems to have been present within the camp in October 2008, with ML2, 4, and 5 all apparently cocirculating over the span of more than a year.

**Fig. 5.—:**
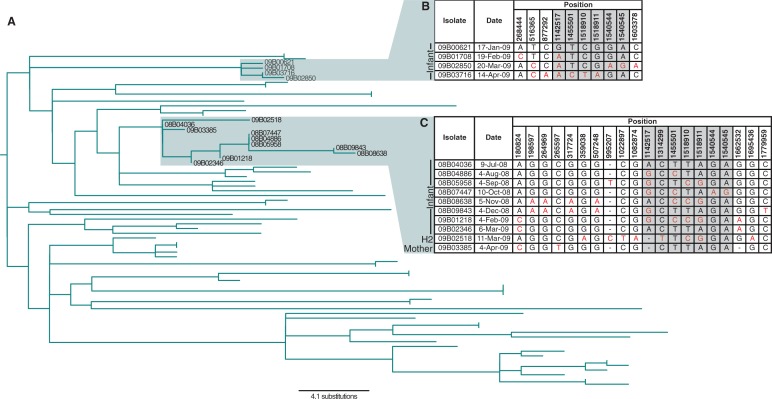
Within-host microevolution and transmission of isolates within the Maela camp. (*A*) The tree represents the clade labeled “ML2” in figure 1, pruned to only include leaf nodes for which complete information on day and site of isolation was available. (*B*) The top inset shows the polymorphic sites identified in four isolates sampled successively from a single infant between January and April 2009, which form a small clade within the overall phylogeny. Positions refer to the reference sequence; shaded columns represent low confidence sites that likely represent false positive signals of variation. Bases are black, when they represent the “ancestral” form found in the first isolate sampled, and red when the bases represent “derived” alleles. Dashes indicate that no bases could be confidently identified at the displayed site. (*C*) The bottom inset shows a clade of ten isolates, eight of which were isolated from a single individual between July 2008 and March 2009, displayed in the same manner as inset (*B*). One isolate (H2) from a nearby household in the same month as this final isolate suggests that a transmission event has occurred between residences; a further isolate from the following month comes from the infant’s mother, indicating a within-household transmission.

Coexistence of diversity could also be detected on a smaller scale. The genomes of eight longitudinally sampled representative colonies from a single individual, isolated between July 2008 and March 2009, revealed 20 polymorphic sites in this population from a single nasopharynx (fig. 5*C*). However, on detailed investigation, seven of these appeared to represent low-quality mapping or phase variation, leaving 13 high-confidence polymorphic sites (nonshaded columns in fig. 5*C*). Little evidence of polymorphism was identified in isolates obtained before October; however, four single-nucleotide polymorphisms were observed to be shared between the isolates from November to December. Subsequently, these polymorphisms were lost from the February and March samples, which shared a third pattern of polymorphisms. The two final members of the clade are from different individuals around the end of the infant’s carriage period: One is from the infant’s mother and the second is from a nearby household, indicating within- and between-household transmission, respectively. A second set of longitudinal samples collected from a single infant between January and April 2009 (fig. 5*B*) again showed that polymorphisms arise and disappear even in the absence of transmission bottlenecks, contrasting with the more clocklike accumulation of diversity over the longer history of the clade.

## Discussion

Whole genome sequencing allows a more precise reconstruction of the emergence and diversification of the PMEN14 clade than afforded by MLST and serotyping. Analysis of this collection reveals that, within ST236, the β–lactam-sensitive 19F isolates represent the persistence of the ancestral phenotype, from which the MDR isolates were derived; subsequently, the β–lactam-sensitive ST236 19A isolates were themselves derived from this MDR genotype. Similarly, both ST271 and ST352 are SLVs of ST236; despite the former differing in serotype, it lies within the PMEN14 clade, whereas ST352 represents an independent emergence of the MDR phenotype. One step further removed, both ST320 and ST202 are serotype 19A DLVs of ST236; yet the former represents a third switch to 19A within the PMEN14 clade, whereas ST202 has independently acquired the 19A capsule, as well as resistance to tetracycline, sulphamethoxazole, and trimethoprim, and is quite distantly related to PMEN14.

The requirement for detailed genetic information to resolve these ambiguities stems from the emergence of the same traits in parallel across the collection. This is typical of “soft” selective sweeps affecting the population (Hermisson and Pennings 2005). These are characterized by multiple independent origins of beneficial alleles emerging in parallel, resulting in more of the ancestral diversity being preserved than in “hard” sweeps where the beneficial mutation has a single origin. Soft sweeps are more likely when the rate of diversification and effective size of the population is high, as in the case of this successful, rapidly recombining lineage. Such behavior can also be facilitated by substructuring, as may be the case for such a geographically disparate population (Pennings and Hermisson 2006). Yet in this collection, evidence was found of differing genotypes coexisting at the scale of a single episode of carriage, based on the disappearance and reappearance of particular alleles from longitudinal sampling. Much greater genetic diversity was found to cocirculate within a single camp with an area of just 4 km^2^ (Turner et al. 2012), some of which impacted on antibiotic resistance phenotypes as a consequence of the observed diversity of macrolide resistance cassettes, *dyr* sequences, and *folP* alleles.

Across the wider collection, each of the resistances observed—to β-lactams, sulpha drugs, tetracycline, and macrolides—were acquired more than once in this sample of a single lineage. This suggests that the emergence of the MDR phenotype may be the product of an ongoing soft selective sweep. Similarly, following the introduction of PCV7, the 19A capsule is observed to be acquired on three independent occasions within the PMEN14 clade alone. However, more epidemiologically rigorous samples of post-PCV7 population structures (Moore et al. 2008; Mahjoub-Messai et al. 2009; Song et al. 2009; Beall et al. 2011; Hanage, Bishop, Lee, et al. 2011) and the MLST database itself (Aanensen and Spratt 2005) suggest that although the initial selective sweeps themselves may be soft, subsequent competition leads to one mutant genotype prevailing. Generally, it seems that the more sparse the sampling or the longer the time between the selective pressure being exerted and samples being collected, the greater the tendency to infer that a genotype has been successful as the lone example of a rare mutation. More focussed data sets may reveal that the genotype has actually had to out-compete others sharing similar mutations selected by the initial sweep, suggesting more stringent selection on the ultimately successful genotype than would otherwise be expected following a strict “bottleneck.”

Of the diversity represented in this collection, the serotype 19A ST320 genotype has been the most successful in the US post-PCV7. This seems partially contingent on PMEN14’s pre-PCV7 success relative to the MDR ST352 genotype, the reason for which is difficult to establish given this current data set; the extensive recombination distinguishing these genotypes may have resulted in selectively important differences, or it could be the consequence of chance founder effects. In the case of the subsequent post-PCV7 competition between different backgrounds having acquired the 19A capsule, it is tempting to focus on the *cps* locus itself. The success of ST320 may stem from it being the only instance within PMEN14 where the entire *cps* locus is replaced, whereas high-level β-lactam resistance is retained. The reduction in resistance of the ST236 19A isolate is likely to be selected against. The mosaic *cps* locus found in the ST271 19A clade may also be less “fit” than the intact 19F or 19A loci, if the cohesion of *cps* loci is driven by epistasis between different genes within the cluster; this would account for its rarity in the United States in 2005, and absence from later samples (Beall et al. 2011). Nevertheless, the observation of these unsuccessful switches to 19A occurring in parallel is interesting in the absence of recombinants having acquired one of the many other common non-PCV7 serotypes, suggesting that serotype switching is nonrandom.

In conclusion, the dense sampling in this analysis indicates that a diverse population of PMEN14, formed through extensive homologous recombination, can coexist even within a small community. This standing variation results in a soft selective sweep in response to a change in selection pressure, which can be observed by deep sampling of a lineage. Subsequent competition between the genotypes that persist after the initial sweep may then result in a single, successful predominant genotype that makes the sweep appear hard. This competition after the intial sweep is likely to represent an important step in selecting the fittest mutants that rise to prominence following the introduction of clinical interventions.

## Supplementary Material

Supplementary figures S1–S6 and table S1 are available at *Genome Biology and Evolution* online (http://www.gbe.oxfordjournals.org/).

Supplementary Data
