# Supplementary material for: Evidence for Soft Selective Sweeps in the Evolution of Pneumococcal Multidrug Resistance and Vaccine Escape
Source: Genome Biol Evol. 2014 Jun 10;6(7):1589–602. doi: 10.1093/gbe/evu120 (PMC4122920; doi:10.1093/gbe/evu120)
Supplement: Supplementary Data [file supp_6_7_1589__index.html]

Evidence for soft selective sweeps in the evolution of pneumococcal multidrug-resistance and vaccine escape — Evidence for Soft Selective Sweeps in the Evolution of Pneumococcal Multidrug Resistance and Vaccine Escape — Supplementary Data 

# Evidence for Soft Selective Sweeps in the Evolution of Pneumococcal Multidrug Resistance and Vaccine Escape

## Supplementary Data

files

**Files in this Data Supplement:**

- Supplementary Data - xlsx file
- Supplementary Data - docx file
