# Supplementary material for: Evidence for Soft Selective Sweeps in the Evolution of Pneumococcal Multidrug Resistance and Vaccine Escape
Source: Genome Biol Evol. 2014 Jun 10;6(7):1589–602. doi: 10.1093/gbe/evu120 (PMC4122920; doi:10.1093/gbe/evu120)
Supplement: Supplementary Data [file supp_evu120_revised.PMEN14_Supplementary_Materials.clean.docx]

# Supplemental Material

**Table S1 – Epidemiological information regarding isolates and accession codes for raw data**

Information regarding the source of isolates, their phenotypic properties, and the raw sequence data used in this analysis. Resistance to antibiotics is detailed where the information is available; numerical values indicate a minimum inhibitory concentration in milligrams per litre, whereas the outcome of phenotypic tests is indicated by the terms ‘sensitive’, ‘intermediate’ or ‘resistant’.


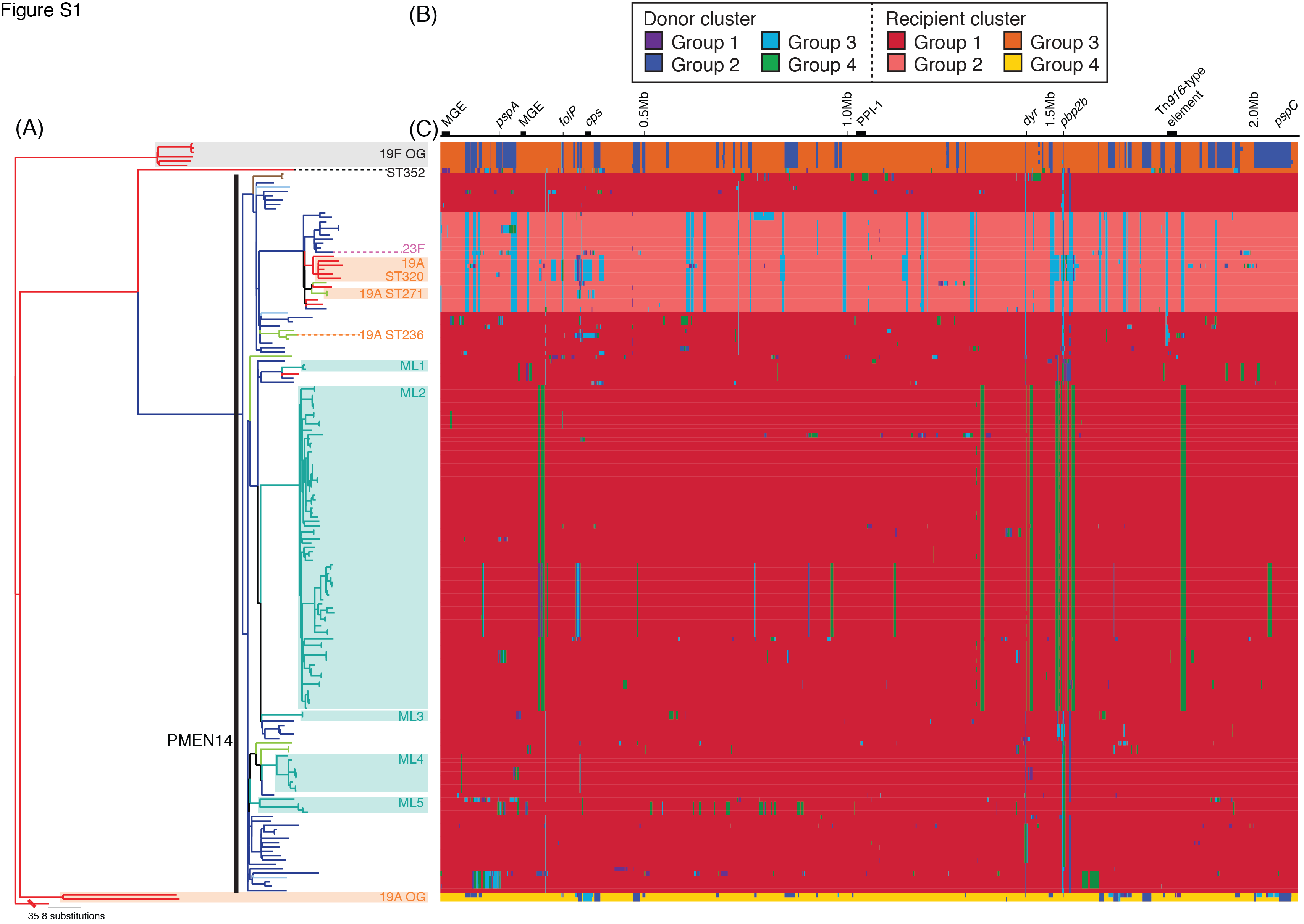


**Figure S1 - BRATNextGen analysis of whole genome alignment.**

The same whole genome alignment used to generate the results displayed in Figure 1 was analysed using BRATNextGen. (A) The maximum likelihood phylogeny, as displayed in Figure 1. (B) The simplified annotation of the reference genome, as displayed in Figure 1. (C) The results of the BRATNextGen analysis. The panel represents one row for each isolate in the phylogeny, with one column for each base in the reference sequence. The background colour of each row represents the recipient cluster to which each isolate belongs, as indicated by the key. Putative recombination events are indicated by blocks coloured according to the predicted donor cluster. This identifies a similar pattern of recombination among the sequences as is displayed in Figure 1, demonstrating the robustness of the analysis to the algorithm used to detect horizontal movement of sequence.

###
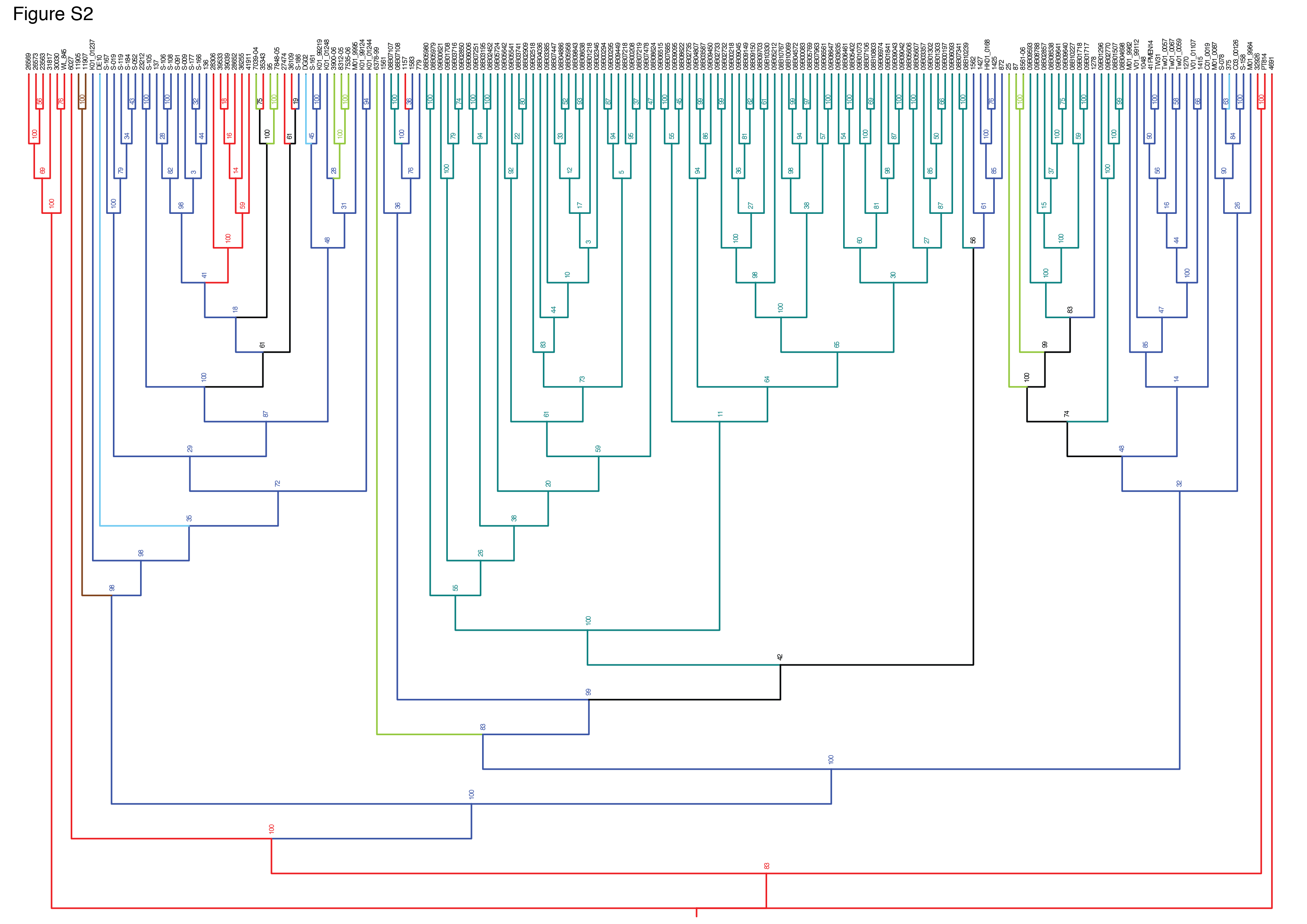


### Figure S2 - Detail of the maximum likelihood phylogeny.

The maximum likelihood phylogeny displayed in Figure 1 is represented as a cladogram, with each leaf node annotated with the isolate name and each internal node annotated with its support, as calculated from 100 bootstraps.


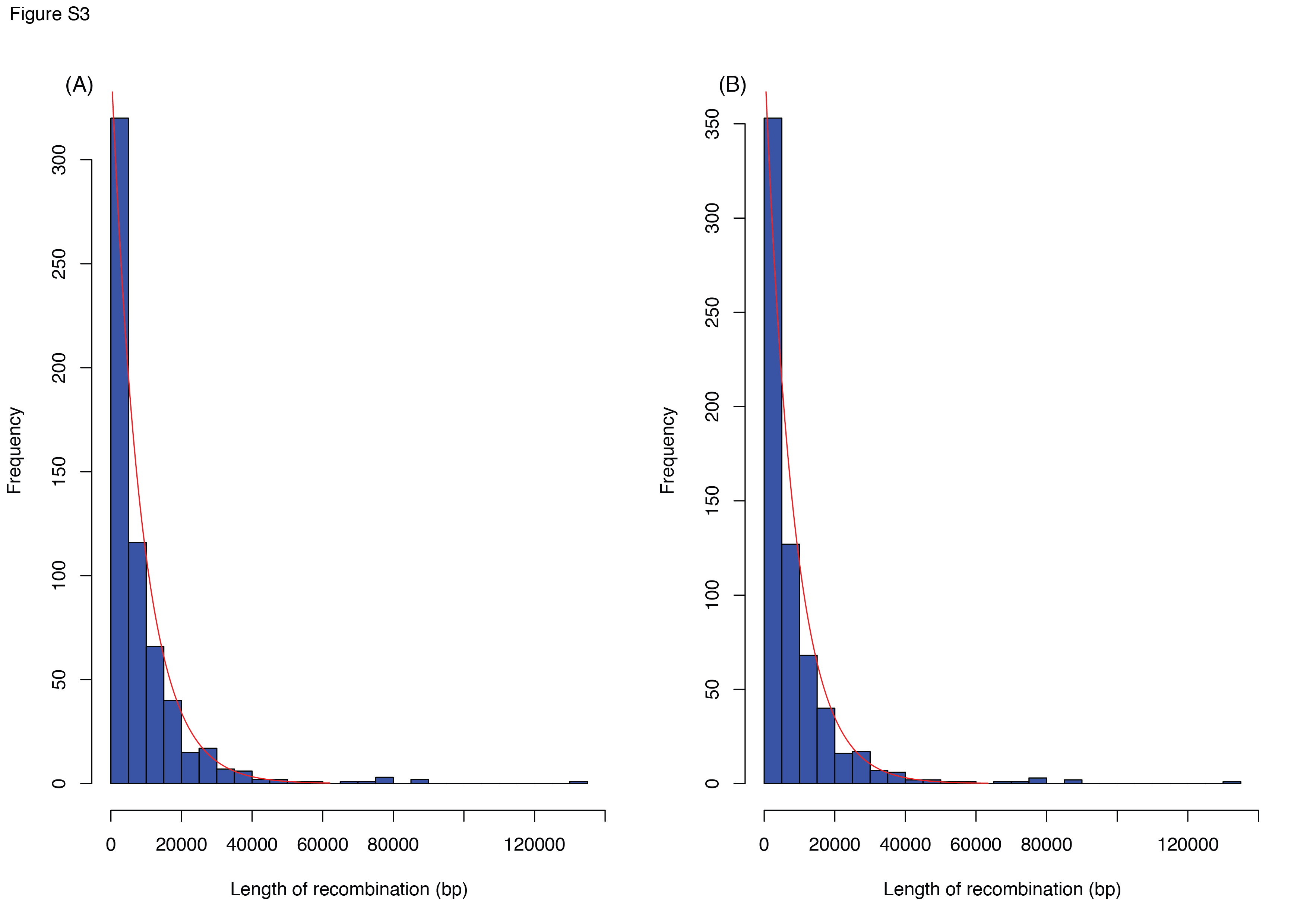


### Figure S3 - Lengths of putative recombination events.

The lengths of recombinations occurring outside of the mobile genetic elements annotated in Figure 1 in (A) the whole collection and (B) only the PMEN14 clade. The actual data, displayed as a histogram, can be approximated by an exponential distribution in both cases, indicated by the red line: the rate parameter estimate in (A) is 1.16x10^-4^ bp^-1^ (95% confidence interval 1.07x10^-4^-1.27x10^-4^ bp^-1^) and in (B) is 1.21x10^-4^ bp^-1^ (95% confidence interval 1.12x10^-4^-1.30x10^-4^ bp^-1^).


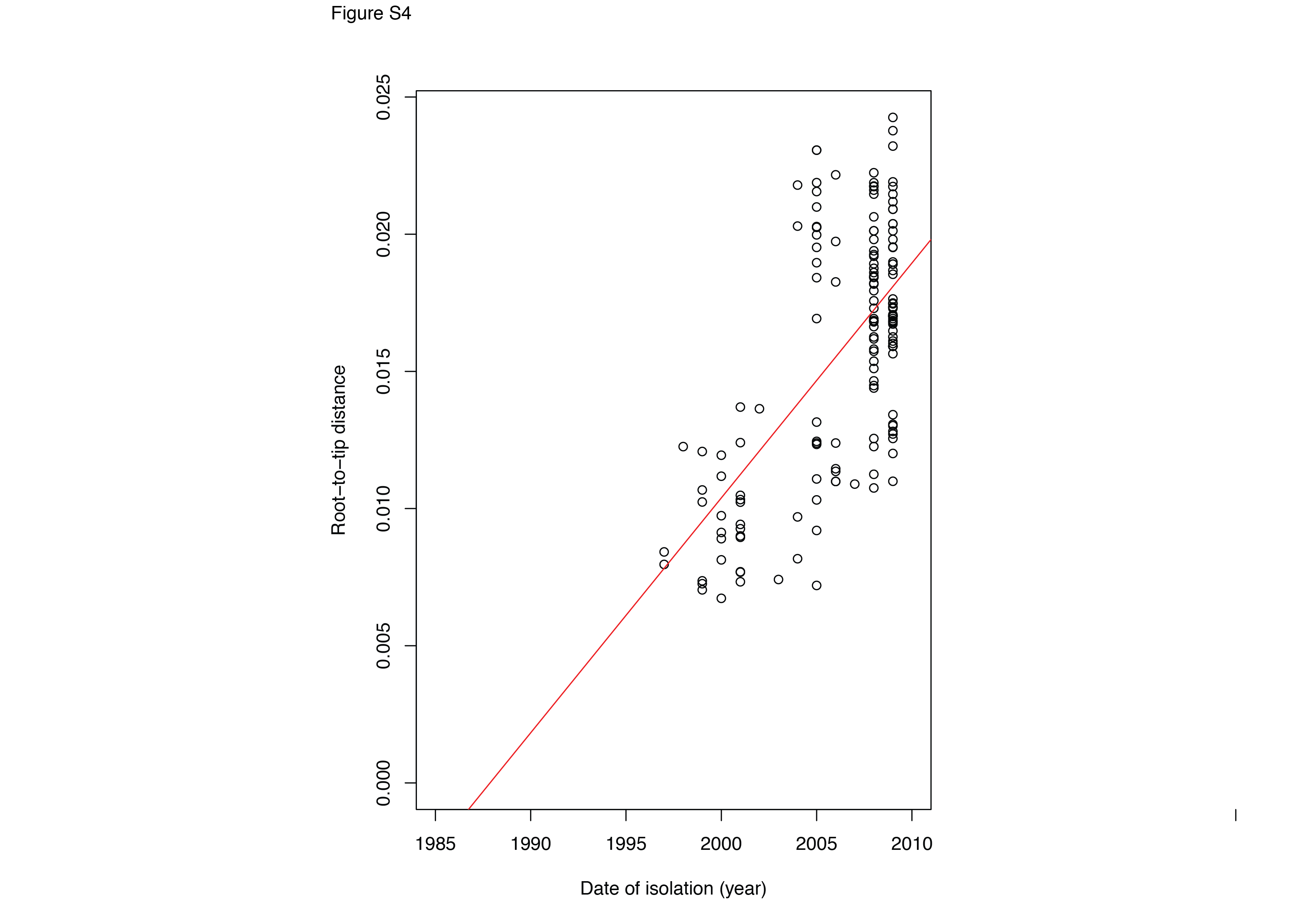


### Figure S4 - Root-to-tip distance plots.

The PMEN14 clade was extracted from the maximum likelihood phylogeny displayed in Figure 1. The distance of each leaf taxon from the root of the tree was plotted against its year of isolation, excluding samples for which no such date was available. This reveals a significant linear correlation coefficient (*R*^2^ = 0.40, n = 164, *p* < 2.2x10^-16^), that implies a date of origin around 1988.

###
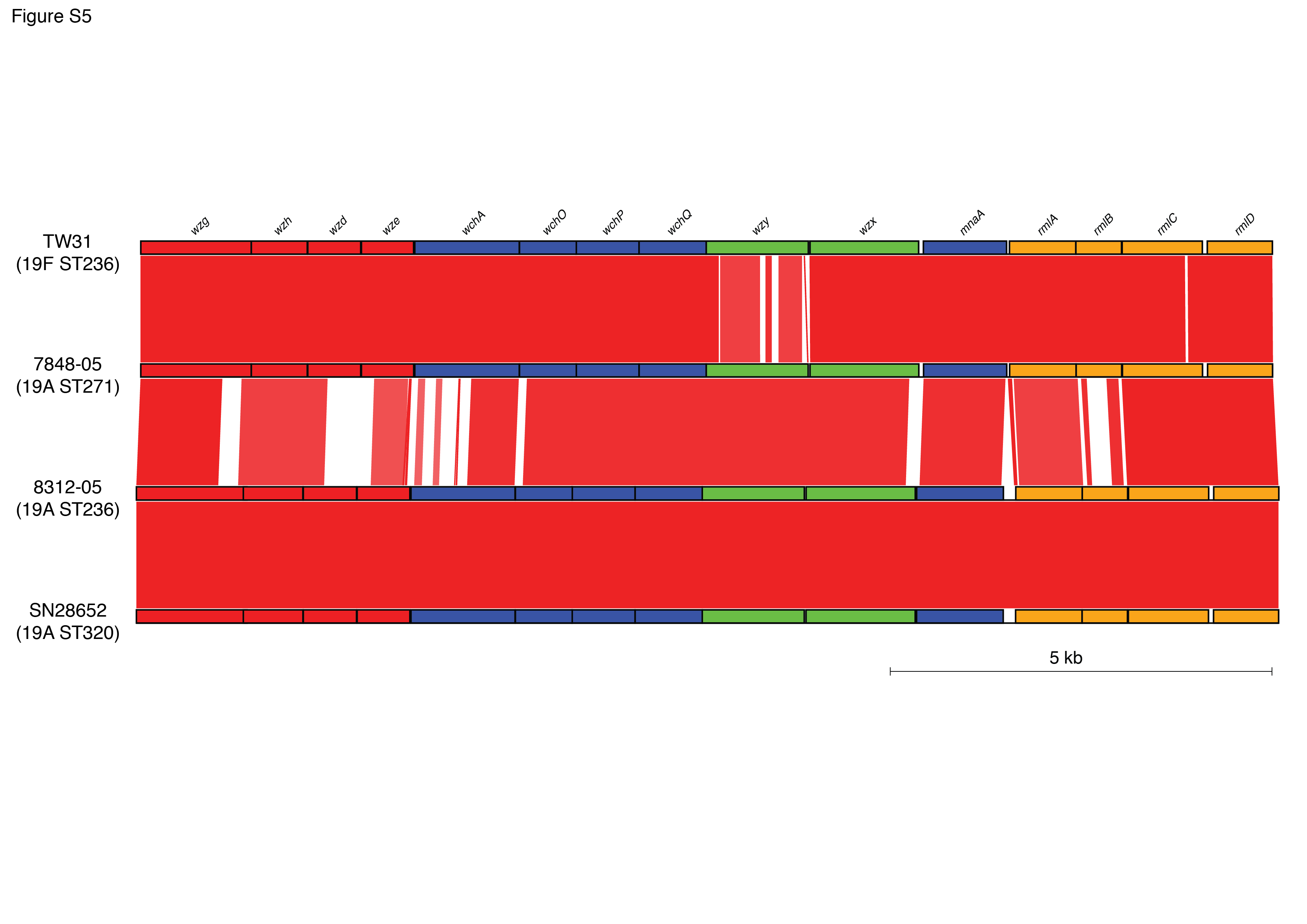


### Figure S5 - Comparison of PMEN14 capsule biosynthesis loci.

The capsule biosyntheis (*cps*) locus of the serotype 19F reference sequence, TW31, is compared with the three serotype 19A *cps* loci acquired within the PMEN14 clade. Protein coding sequences are coloured red, if encoding regulatory functions, blue if encoding glycosyltransferases involved in monomer synthesis, green if involved in polymerisation or transport, and orange if involved in rhmanose biosynthesis. The red bands between the sequences indicate BLAT matches, with the strength of the colour indicating the degree of similarity between the sequences. This shows that the 19A *cps* locus of isolate 7848-05 strongly differs from that of the reference genome only at the *wzy* polymerase gene. The other 19A *cps* loci differ from that of 7848-05 by divergence in *wchA*, the genes encoding regulatory functions and the rhamnose biosynthesis gene operon.

###
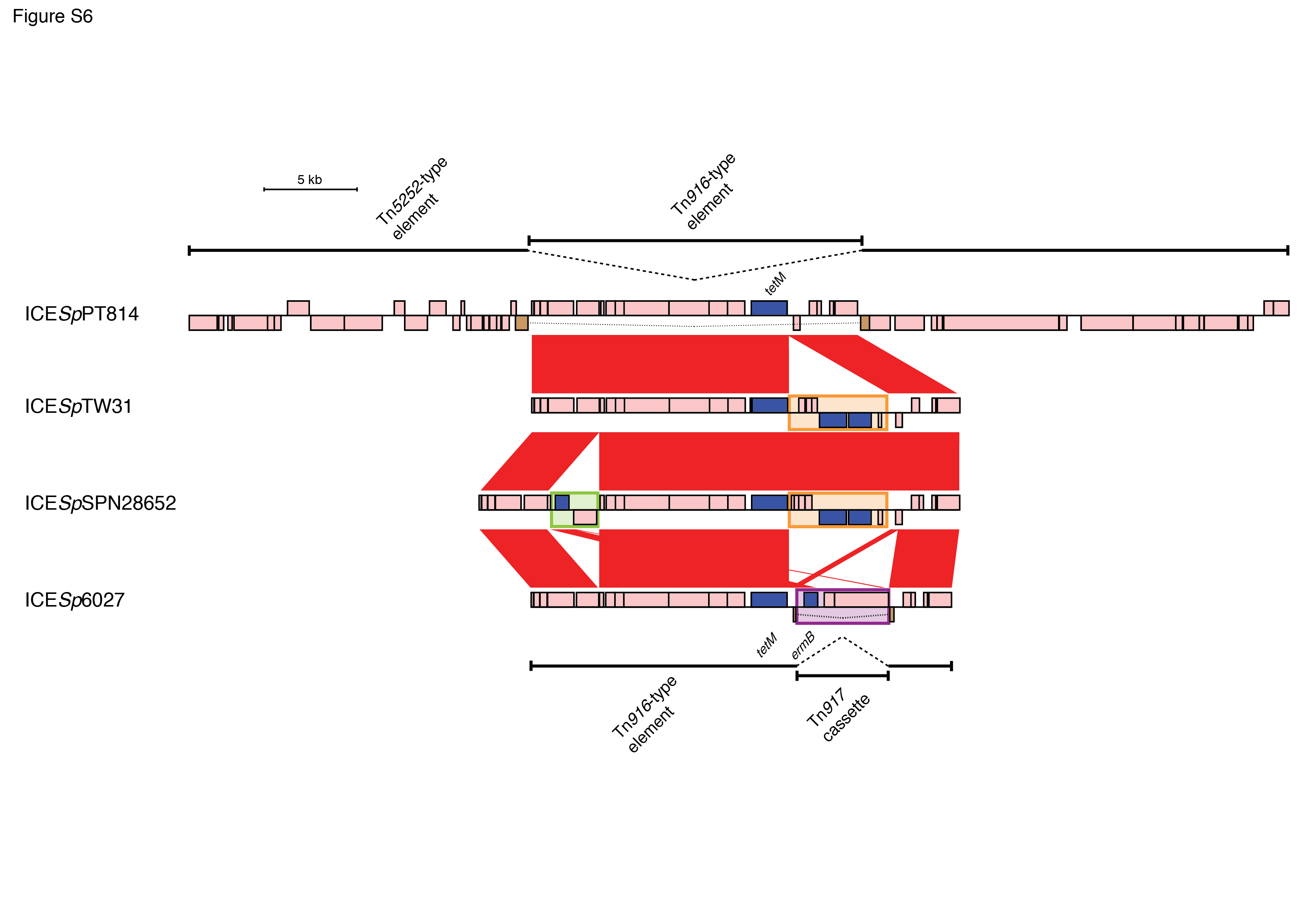


### Figure S6 - Integrative and conjugative elements present in the collection.

The integrative and conjugative elements (ICE) found in the collection are aligned to show the independent acquisition of different resistance genes. Each ICE is extracted from the *de novo* assembly of an individual isolate. The coding sequences (CDSs) are annotated as pink boxes (except antibiotic resistance genes, which are coloured blue), with the position above or below the central line representing whether the CDS is found on the forward or reverse strand of the sequence. The red bands between the sequences indicate BLAT matches, with the strength of the colour indicating the degree of similarity between the sequences. ICE*Sp*PT814 is a large Tn*5253*-type element composed of a shorter Tn*916*-type component inserted into a large Tn*5252*-type sequence. The insertion of the Tn*916*-type element splits a protease gene, indicated by the brown boxes linked by a dashed line. The *tetM* tetracycline resistance gene is annotated. ICE*Sp*TW31 is from the reference sequence, and consists of a Mega element (boxed in orange) inserted into a Tn*916*-type sequence. This is likely to be the form originally acquired by PMEN14; however, the clade that encompasses ST271 and ST320 isolates has the ICE represented by ICE*Sp*SPN28652, which also includes an *ermB* macrolide resistance gene carried on an Omega cassette fragment (boxed in green). The ST352 isolate 6027 has the alternative ICE*Sp*6027, which instead carries the *ermB* resistance gene on a Tn*917* cassette (boxed in purple) within the Tn*916*-type transposon.
